# Interplay between enterohaemorrhagic *Escherichia coli* and nitric oxide during the infectious process

**DOI:** 10.1080/22221751.2020.1768804

**Published:** 2020-05-27

**Authors:** Ilham Naïli, Marion Gardette, Annie Garrivier, Julien Daniel, Mickaël Desvaux, Mariagrazia Pizza, Alain Gobert, Thierry Marchal, Estelle Loukiadis, Grégory Jubelin

**Affiliations:** a Université Clermont Auvergne, INRAE, MEDiS, F-63000 Clermont-Ferrand, France; b GSK, Siena, Italy; c Université de Lyon, CNRS, INRAE, Université Claude Bernard Lyon 1, VetAgro Sup, Laboratoire d'Ecologie Microbienne, F-63280 Marcy l'Etoile, France; d VetAgro Sup, Laboratoire vétérinaire d'histopathologie, F-63280 Marcy-l'Etoile, France; e VetAgro Sup, Laboratoire national de référence des E. coli, F-63280 Marcy-l'Etoile, France

**Keywords:** Enterohaemorrhagic *E. coli*, nitric oxide, Shiga toxins, gut pathogen, mouse models of infection

## Abstract

Enterohaemorrhagic *Escherichia coli* (EHEC) are bacterial pathogens responsible for life-threatening diseases in humans such as bloody diarrhoea and the hemolytic and uremic syndrome. To date, no specific therapy is available and treatments remain essentially symptomatic. In recent years, we demonstrated *in vitro* that nitric oxide (NO), a major mediator of the intestinal immune response, strongly represses the synthesis of the two cardinal virulence factors in EHEC, namely Shiga toxins (Stx) and the type III secretion system, suggesting NO has a great potential to protect against EHEC infection. In this study, we investigated the interplay between NO and EHEC *in vivo* using mouse models of infection. Using a NO-sensing reporter strain, we determined that EHEC sense NO in the gut of infected mice. Treatment of infected mice with a specific NOS inhibitor increased EHEC adhesion to the colonic mucosa but unexpectedly decreased Stx activity in the gastrointestinal tract, protecting mice from renal failure. Taken together, our data indicate that NO can have both beneficial and detrimental consequences on the outcome of an EHEC infection, and underline the importance of *in vivo* studies to increase our knowledge in host–pathogen interactions.

## Introduction

Enterohaemorrhagic *E. coli* (EHEC), especially those belonging to the O157:H7 serotype, are foodborne pathogens responsible for intestinal disorders that may ultimately evolve to life-threatening diseases such as the hemolytic uremic syndrome (HUS) or the thrombotic thrombocytopenic purpura. To date, no specific therapy is available to fight EHEC infections in humans since antibiotics are usually not recommended. Treatments remain essentially symptomatic and most patients require prolonged clinical and follow-up outpatient care [1,2]. Healthcare costs associated with such disease are thus very significant [3]. A range of virulence factors are involved in EHEC pathogenicity [4,5]. This includes the type III secretion system (T3SS), which enables the pathogen to attach to the intestinal epithelium and causes the formation of characteristic attaching and effacing lesions at the surface of enterocytes [6]. The T3SS is a protein-based complex resembling a molecular syringe that allows injection of bacterial effectors into epithelial cells where they subvert specific cell signalling pathways [7]. Among them, the protein Tir becomes inserted into the host cell membrane and acts as a receptor for the intimin protein (encoded by the *eae* gene) localized at the bacterial surface, leading to an intimate adhesion between bacteria and enterocytes. Genes encoding the T3SS are gathered into five major operons located into a pathogenicity island called the locus of enterocyte effacement (LEE). In addition to the T3SS, EHEC virulence is closely associated with the production of Shiga toxins (Stx). Two antigenically distinct forms of Stx, Stx1 and Stx2, can be produced alone or in combination by EHEC strains [8]. Epidemiological studies have shown that Stx2-producing *E. coli* strains are however more likely to cause HUS than strains producing Stx1 only [9]. Stx-encoding genes are located in lambdoid phages integrated into the bacterial chromosome and their expression is mainly driven by activation of the SOS response in response to environmental cues [10]. Once produced in the gut lumen, Stx translocates across the intestinal epithelium, reaches the bloodstream and targets cells expressing the receptor glycolipid globotriaosylceramide-3 (Gb3). Upon internalization and retrograde transport, Stx alters ribosomal function and induces cell necrosis or apoptosis [11]. The kidneys and brain are the two main target organs that suffer from Stx intoxication in EHEC-infected patients, leading to the development of diseases such as HUS. Since Stx diffuses via the bloodstream and can theoretically affect the vasculature of every organ, specific injuries of the kidneys and brain in HUS patients may be explained by cell-specific variations of Gb3 expression [12]. In the kidneys, glomerular endothelial and mesangial cells as well as tubular epithelial cells highly express Gb3 and are sensitive to Stx [2,13] and their intoxication contributes to the development of thrombotic microangiopathic lesions.

Intestinal pathogens, including EHEC, first have to reach and adapt to the gut environment before producing their virulence factors and causing disease [4]. Among the harmful conditions imposed by the gastrointestinal tract, pathogens must survive and adapt to oxidative and nitrosative stresses. These include exposure to reactive oxygen species (ROS) and reactive nitrogen species (RNS) liberated by mucosal immune cells in response to infection. A key component in RNS synthesis by the host is nitric oxide (NO), a highly reactive inorganic free radical produced from L-arginine by NO synthases (NOS). NO can react with a large spectrum of molecules such as inorganic elements, various DNA structures, proteins and lipids, thereby carrying a strong antimicrobial activity [14]. To overcome this aggression, intestinal pathogens such as pathogenic *E. coli*, *Salmonella* spp. and *Campylobacter jejunii*, have developed numerous mechanisms involved in NO detoxification [15]. These notably include globins, which oxidize NO into nitrate, and reductases which reduce NO into nitrous oxide. Inactivation of these proteins leads to mutants with higher susceptibility to NO, impaired survival within macrophages and reduced virulence capacity in their respective host [16–18]. We have previously demonstrated that subinhibitory concentrations of NO inhibit the synthesis of Stx2 by EHEC and the release of Stx2 phages via inhibition of the SOS response *in vitro* [19]. We also have shown that NO represses the expression of several LEE genes and, consequently, drastically reduces EHEC adhesion to epithelial cells *in vitro* [20]. Both effects are mediated by the NO responsive regulatory protein NsrR. By repressing the production of the two major virulence factors in EHEC, combined with its typical antimicrobial effect, NO has therefore a great potential to protect against an EHEC infection. In this context, the aim of this work was to decipher the role of NO in the control of EHEC *in vivo* using mouse models of infection. First, we determined that EHEC sense NO in the gut of infected mice. Using a specific NOS inhibitor, we next demonstrated that host-produced NO inhibits EHEC adhesion to colonic mucosa but increases Stx toxicity in the gastrointestinal tract. We also showed that inhibition of NOS activity rescues EHEC-infected mice from developing kidney failure. Taken together, our data indicate that NO can have both beneficial and detrimental consequences in the course of an EHEC infection.

## Materials and methods

### Bacterial strains, plasmids and growth conditions

Strains and plasmids used in this study are listed in Table S1. A streptomycin resistant derivative of the prototype EHEC strain EDL933 was used throughout the study. All strains were grown in LB medium at 37°C unless otherwise indicated. When required, antibiotics were used at the following concentrations: 25 µg.ml^−1^ kanamycin; 50 µg.ml^−1^ ampicillin; 25 µg.ml^−1^ chloramphenicol; 15 µg.ml^−1^ gentamycin; 50 µg.ml^−1^ streptomycin (Sm).

### Construction of a NO-sensing EHEC reporter strain

In order to monitor NO sensing by EHEC, we constructed a reporter strain using the TnpR recombinase-based *in vivo* expression technology (RIVET) [21]. To this end, the promoter region of gene *ytfE*, known to be highly induced by NO in *E. coli* [22], was amplified from genomic DNA of EDL933 using primers 2F10-F (5’-CTGCAGGAAGATCTGTGGTCATCGCGGTTAGAGC-3’) and 2F10-R (5’-GATCGTGAAGATCTCGATAAGCCATAGCTGATACCTCATTC-3’), which contain *Bgl*II restriction sites in their 5’ ends. The PCR product and vector pGOA1193 were digested with *Bgl*II and ligated to yield plasmid p1193-*ytfE* carrying a P*_ytfE_*-*tnpR* transcriptional fusion. Correct orientation of *ytfE* promoter was validated by PCR and sequencing. The plasmid was mobilized through a mating experiment into EDL-RES, an EDL933 strain harbouring the RES-flanked marker cassette that contains a gene conferring kanamycin resistance and a gene conferring sucrose sensitivity [23]. Plasmid insertion into the *ytfE* locus was verified by PCR and sequencing.

### Resolution assays

To validate the ability of our reporter strain to detect NO, EDL-RES P*_ytfE_*-*tnpR* strain was grown for 6 h in LB supplemented or not with increasing concentrations of NOR-4, a NO donor (Enzo Life Sciences). Expression of TnpR was monitored by calculating the percentage of bacteria that have lost the RES marker cassette. This percentage, termed resolution, is calculated for individual samples by dividing the titre obtained on LB agar plates without NaCl, with Sm and 4% sucrose (resolved bacteria) by the titer obtained on LB agar with Sm (total bacteria). Resolutions were also calculated from faecal samples of mice infected with EDL-RES P*_ytfE_*-*tnpR* to evaluate the sensing of NO by EHEC *in vivo* during infection.

### Mouse infection

All animal experiments were reviewed and approved by the Auvergne Committee for Animal Experimentation C2EA (Agreement N°7289-2016093010075533). C57BL/6 female mice 5 weeks old with SPF status were purchased from Janvier Labs (Le-Genest-St-Isle, France) and housed in cages of no more than five mice per cage. Mouse experiments were performed with 5–10 mice per group and repeated on (at least) two separate occasions. Mice were given drinking water containing 5 g/L of streptomycin sulphate (Sigma-Aldrich) throughout the experiment. For some groups, drinking water was also supplemented with 1 g/L of N^ω^-nitro-L-arginine methyl ester hydrochloride (L-NAME; Enzo Life Science) and changed daily over the course of the experiment. On day 1 following the addition of streptomycin, each mouse was infected intragastrically with 100 µl of PBS containing 10^7^ bacterial cells of EDL933 Sm^R^ grown in LB for 6 h. Uninfected mice were given PBS only. At indicated time points, faecal samples were collected, homogenized in PBS and subsequently diluted before plating on LB + streptomycin agar plates or Stx activity quantification. To quantify adherent bacteria to the gut mucosa, a piece of both caecum and colon were taken at day 7, cut longitudinally and washed extensively in PBS before dilution and plating. Adhesion was expressed as the ratio between adherent bacteria and the total number of EHEC detected in faecal samples at the same time point. For the lethal infection model, mice also received an intraperitoneal dose of 40 µg of ciprofloxacin (prepared in 500 µl PBS) 1, 2 and 3 days post-infection (DPI) (adapted from [24]). Body weight and clinical signs of mice were monitored daily to evaluate the severity of infection. Mice presenting a weight loss > 15% compared to body weight at day 0, or presenting severe clinical symptoms such as ataxia and/or lethargy, were immediately euthanized. Urine was collected daily for determination of the specific gravity using a refractometer, as well as urea concentration using the QuantiChrom Urea assay kit (BioAssay Systems).

### Histological analyses

Kidneys were excised from euthanized mice at 7 DPI and were immediately fixed in PBS + formol 4% (Neutral Buffered Formalin, Diapath) during 48 h before being embedded in paraffin. Tissue sections were stained with haematoxylin and eosin. The sections were examined for glomerular, tubular, interstitial and vascular changes by light microscopy. Images were acquired and analysed using Caseviewer software.

### Quantification of Stx activity

The Stx activity from different samples was monitored using the Vero-d2EGFP cell line [25]. The cell line was maintained and propagated routinely at 37 °C with 5% CO_2_ under humidified conditions in a complete medium made of DMEM supplemented with 10% fetal bovine serum (Gibco), Zell Shield (Minerva Biolabs) and 200 µg.ml^−1^ Geneticin (Gibco). For the assays, Vero-d2EGFP cells were seeded in black 96-well plates with clear bottoms at 3.10^4^ cells per well and incubated for 3 days to reach 80–90% confluence. Samples to be tested as well as purified Stx2 (Toxin Technology) used as an internal standard were two-fold diluted in complete medium, then transferred to Vero-d2EGFP-containing plates. After a 16 h incubation period in a CO_2_ incubator, samples were removed and 100 µl of PBS was added to each well before GFP quantification in a Spark microplate reader (Tecan) with excitation at 485 ± 20 nm and emission at 530 ± 20 nm. Stx activity was expressed as an arbitrary unit by comparing fluorescence values from samples with the standard curve obtained with purified Stx2.

### Quantification of mRNA

Frozen colon section from different mice was crushed in liquid nitrogen and total RNA was isolated using TRIzol reagent (Invitrogen), treated with DNAse I (Fermentas), and reverse-transcribed (1 μg) using oligo(dT) primers and 5 U/μl Superscript II reverse transcriptase (Invitrogen). Real-time PCR was performed with the QuantiFast SYBR Green PCR kit (Qiagen) following the manufacturer’s instructions and using 2 µl of cDNA and primers specific to NOS1 (5’-CTGGTGAAGGAACGGGTCAG-3’ and 5’-CCGATCATTGACGGCGAGAAT-3’), NOS2 (5’-TCAGAGCCACAGTCCTCTTT-3’ and 5’-TCCATGCAGACAACCTTGGT-3’), NOS3 (5’-TGTGACCCTCACCGCTACAA-3’ and 5’-GCACAATCCAGGCCCAATC-3’) or β-actin (5’-GGCTGTATTCCCCTCCATCG-3’ and 5’-CCAGTTGGTAACAATGCCATGT-3’). Results were calculated using the comparative cycle threshold method and are expressed as relative mRNA expression compared with the uninfected condition.

### Statistical analyses

All statistics were made using the GraphPad Prism software version 8 (San Diego, California). Unpaired two-tailed student’s *t* tests were used to determine significant differences between two groups. ANOVA with the Holm-Sidak correction or Dunn’s comparisons tests were used to analyse differences among multiple groups. The Log-rank (Mantel–Cox) test was used to compare survival curves. Statistical tests used for each data analysis are indicated in figure legends. *P* < .05 was considered significant.

## Results

### EHEC sense NO in the gut of infected mice

**Figure 1.**
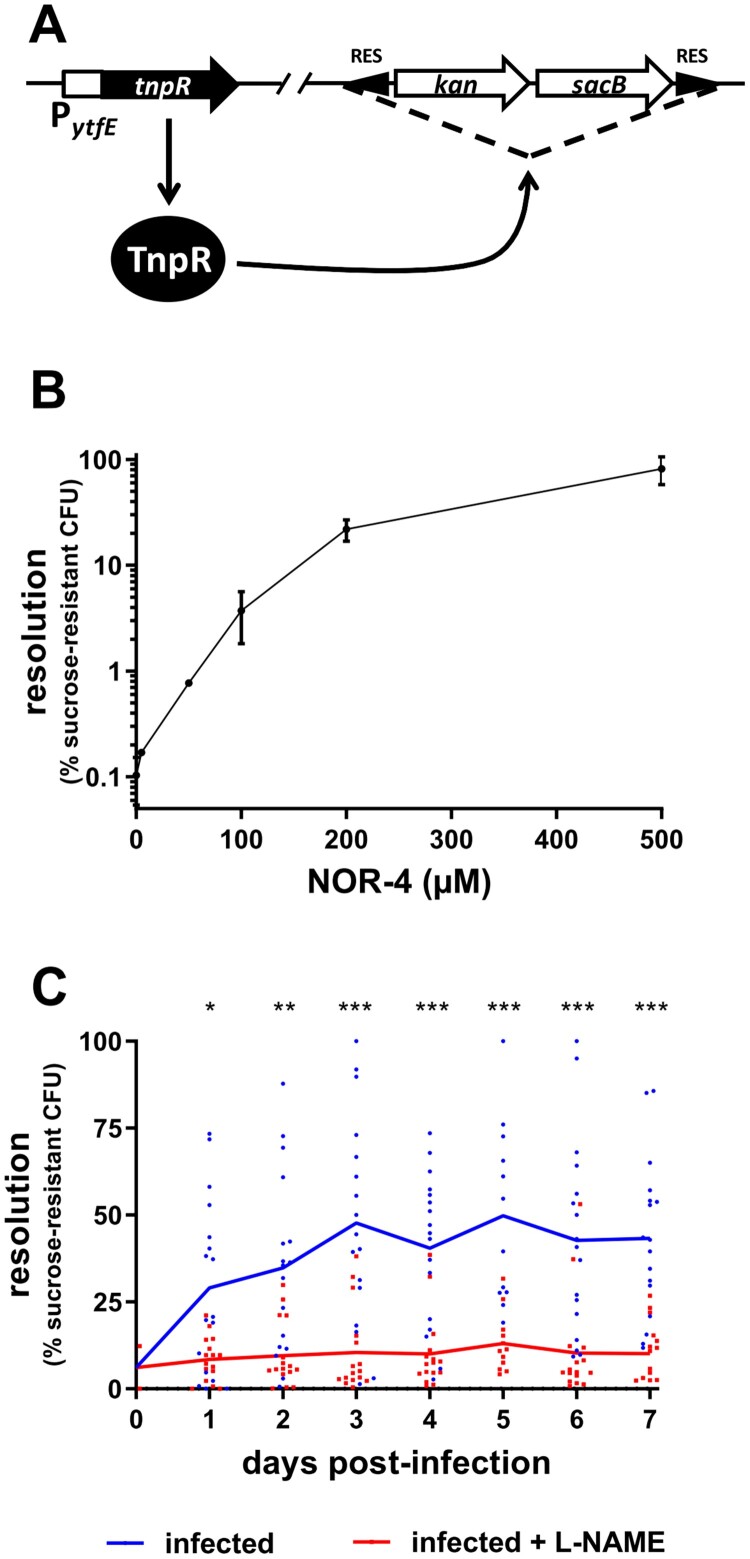
EHEC sense NO in the gut of infected mice. (A) Schematic representation of the NO-sensing reporter system. The EHEC EDL933 strain carries a RES-flanked marker cassette and a P*_ytfE_*-*tnpR* transcriptional fusion. In the presence of NO, P*_ytfE_* is activated, leading to the expression of TnpR recombinase and excision of the cassette. The strain becomes kanamycin-sensitive and sucrose-resistant. (B) The NO reporter strain was grown for 6 h in LB supplemented with various concentrations of NOR-4. Resolutions (percentage of bacteria that have lost the RES marker cassette) were calculated following bacterial numeration on plates with or without sucrose. Values represent the mean ± standard deviation. (C) Mice, treated or not with the NOS inhibitor L-NAME, were infected with the EHEC NO reporter strain. At the indicated time points, resolutions were calculated from faecal samples. Each dot represents one mouse and curves represent mean values. A two-tailed unpaired *t*-test was applied to compare both groups. ns: non-significant; *p <* *.05; **p <* *.01; ***p <* *.001.

Before investigating the role of NO in the control of EHEC infection, we first wanted to evaluate whether EHEC are able to sense NO in the gut of infected mice. To this end, we constructed an EHEC EDL933 strain that reports the detection of NO using elements from the RIVET system [21], a RES cassette containing two selective markers, and the reporter gene *tnpR*, which encodes a recombinase that specifically cleaves the marker cassette (Figure 1(A) and see M&M). The reporter gene was placed under the control of the promoter region of gene *ytfE*, whose expression is known to be highly upregulated in the presence of NO through the release of repressing activity from NsrR, a major NO-sensing regulatory protein in *E. coli* [22,26]. To validate the efficiency of our reporter strain to detect NO, the strain was grown in LB with various concentrations of NOR-4, a NO donor (Figure 1(B)). As expected, the resolution (i.e. the percentage of bacteria that have lost the RES cassette) from a bacterial culture without NOR-4 was very low, indicating that the P*_ytfE_*-*tnpR* fusion was not expressed in the absence of NO. In contrast, the addition of increasing concentrations of NOR-4 led to a strong increase in resolution, reaching more than 80% with 500 µM of NOR-4 (Figure 1(B)). Because deletion of the marker cassette is irreversible, spontaneous cassette deletion event and/or basal expression of the recombinase may lead to an increased resolution over a long time period even in the absence of NO signal. We therefore assessed the behaviour of our reporter strain during a 7-day period time of *in vitro* growth and observed that the resolution level is very stable over time and stayed low in the absence of NO, or in contrast stayed high after an initial NO exposure (Supplementary Figure S1). These data demonstrate that our reporter strain efficiently detects the presence of NO in the surrounding environment. Next, we infected mice with this reporter strain and monitored the resolution status of EHEC recovered from animals’ faeces over a 7-day period time. As shown in Figure 1(C), the resolution was low at the beginning of infection, increased gradually between 1 and 3 days post-infection (DPI) and remained stable until the end of experiment. This result indicates that EDL933 detects NO in the gut during mouse infection. We next infected mice treated with L-NAME, a specific inhibitor of NOS activity. Whereas the gut colonization by EDL933 was unaffected by L-NAME treatment (Supplementary Figure S2), the resolution level recorded from L-NAME treated mice did not increase over time and was significantly lower than the resolution obtained with untreated mice. This indicates that treatment of mice with L-NAME efficiently reduced the level of NO that EHEC encounter in the gut during an infection. L-NAME treatment of mice has been therefore used in the next sections to evaluate the role of host produced NO in the outcome of an EHEC infection.

### NO limits EHEC adhesion to the colonic mucosa but enhances Stx toxicity in the gut

**Figure 2.**
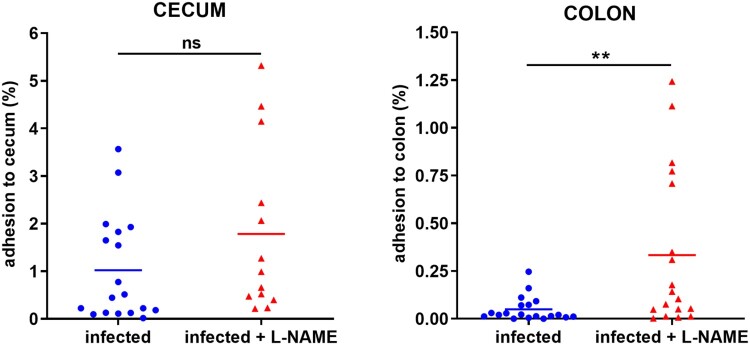
L-NAME treatment of infected mice increases EHEC adhesion to the colonic mucosa. Mice, treated or not with L-NAME, were infected with EDL933 and euthanized at 7 DPI. Caecum and colon were harvested, washed in PBS, crushed and then plated on LB plates with Sm in order to quantify mucosa-associated EHEC. Data are represented as the percentage of adherent bacteria relative to the total number of EHEC quantified in faecal samples. Each dot represents one mouse and means are indicated as a line. A two-tailed unpaired *t*-test was applied to compare both groups. ns: non-significant; **p <* *.01.

**Figure 3.**
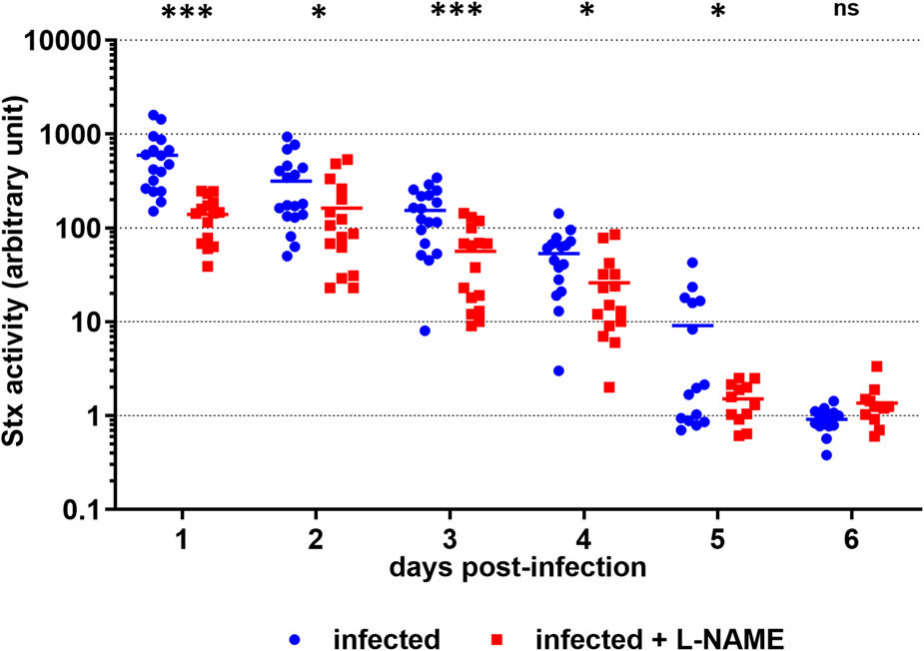
L-NAME treatment of infected mice limits Stx toxicity in the gut. Mice, treated or not with L-NAME, were infected with EDL933. At the indicated time points, Stx activity from faeces was quantified using the Vero-d2EGFP cell line. Each dot represents one mouse and means are indicated as a line. A multiple two-tailed unpaired *t*-test was applied to compare both groups every day. ns: non-significant; *p <* *.05; ***p <* *.001.

We have previously shown that NO inhibits EHEC adhesion to epithelial cells *in vitro* [20]. To assess whether NO affects the ability of EHEC to adhere to the gut epithelium *in vivo*, mice treated or not with L-NAME were infected with EDL933 and adherent bacteria were quantified from caecal and colonic tissues 7 DPI. No difference was observed between the two groups in terms of bacterial adherence to the caecal tissue at 7 DPI (Figure 2). In contrast, the proportion of EHEC adherent to the colon was significantly increased by a factor 7 in mice treated with L-NAME (Figure 2). These data demonstrate that inhibition of NOS activity in infected mice favours EHEC adherence to the colonic epithelium. We have determined that NO also inhibits the production of Stx2 during *in vitro* growth of EDL933 [19]. We therefore investigated whether mouse treatment with L-NAME affects the level of Stx in the gut of EDL933-infected mice. We first tried to quantify Stx2 as previously described [27] but the ELISA method was not sensitive enough to accurately detect Stx in faecal samples collected from infected mice. We next evaluated Stx activity (Stx1 + Stx2) using Vero-d2EGFP, a Stx-sensitive cell line harbouring a destabilized variant of GFP used to monitor protein synthesis inhibition [25]. Stx activity was significantly lower in faeces of infected mice treated with L-NAME versus untreated mice (Figure 3). Indeed, the toxin activity was reduced between 1 and 5 DPI with a decrease fold change ranging from 2 to 6 compared to untreated mice. No Stx activity was detected in urine (day 6) or serum (day 7) of infected mice from either group (data not shown). These data indicate that inhibition of NOS activity in infected mice decrease Stx activity and strongly suggest that host-produced NO favours activity of the toxin during infection.

### NO exacerbates renal failure following a lethal EHEC challenge

**Figure 4.**
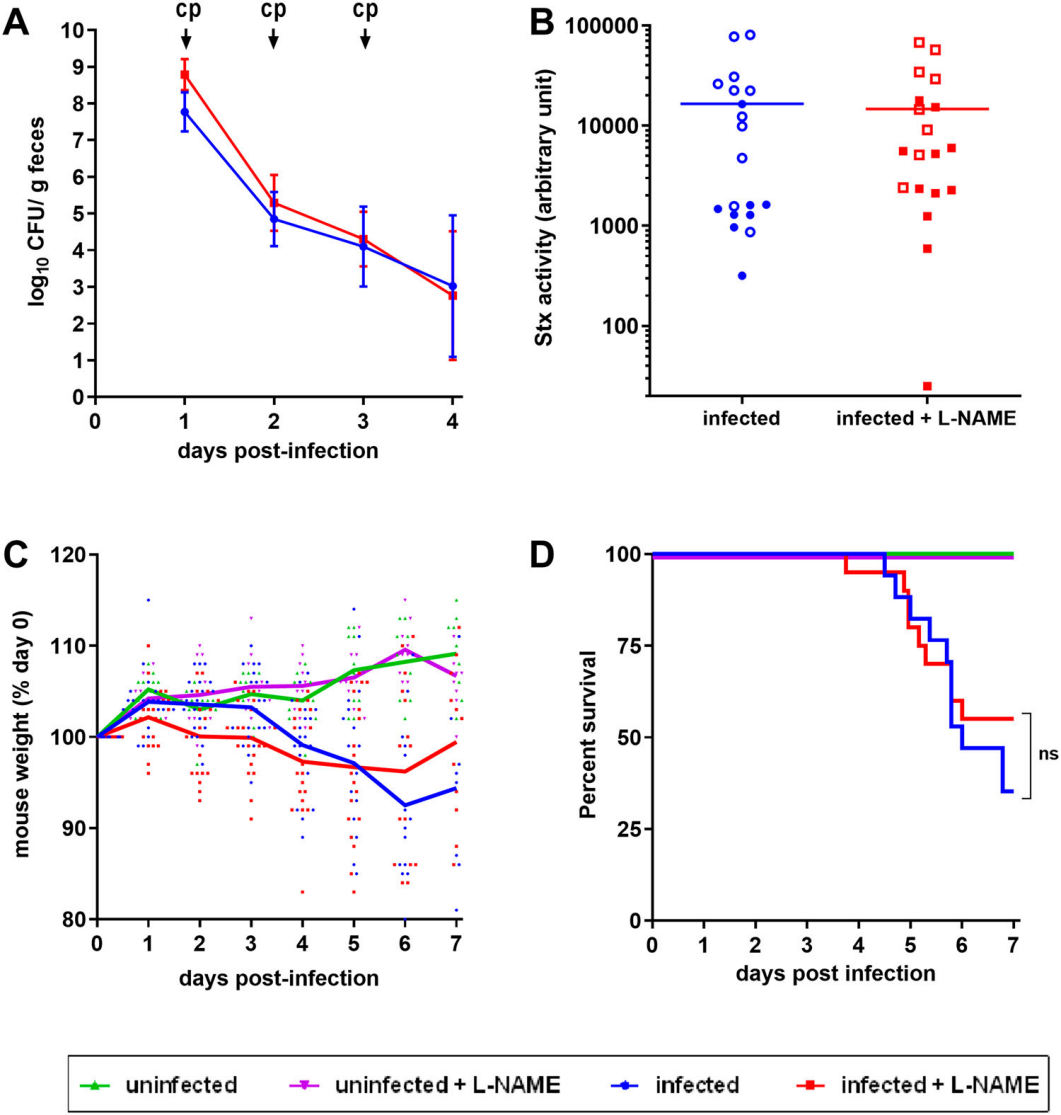
Role of NO in a lethal EHEC infection model. Mice, treated or not with L-NAME, were left uninfected or were infected with EDL933. Each mouse was injected 40 µg of ciprofloxacin (cp) intraperitoneally at 1, 2 and 3 DPI to induce Stx production and release. (A) At the indicated time points, EHEC shedding was quantified by plating faecal samples on LB plates with Sm. Values represent the mean ± standard deviation. (B) Stx activity from faeces was quantified at 2 DPI using Vero-d2EGFP cells. Each dot represents one mouse and means are indicated as a line. Open and closed symbols indicate mice that were dead or alive at 7 DPI, respectively. (C) Mouse weights were recorded every day post-infection and weight curves are presented as the percentage relative to the recorded weight at the day of infection (day 0). Each dot represents one mouse and curves represent mean values. (D) The survival time and rate were recorded for 7 days after infection, and statistic were obtained using a Log-rank (Mantel–Cox) test (*n* = 20 per group).

**Figure 5.**
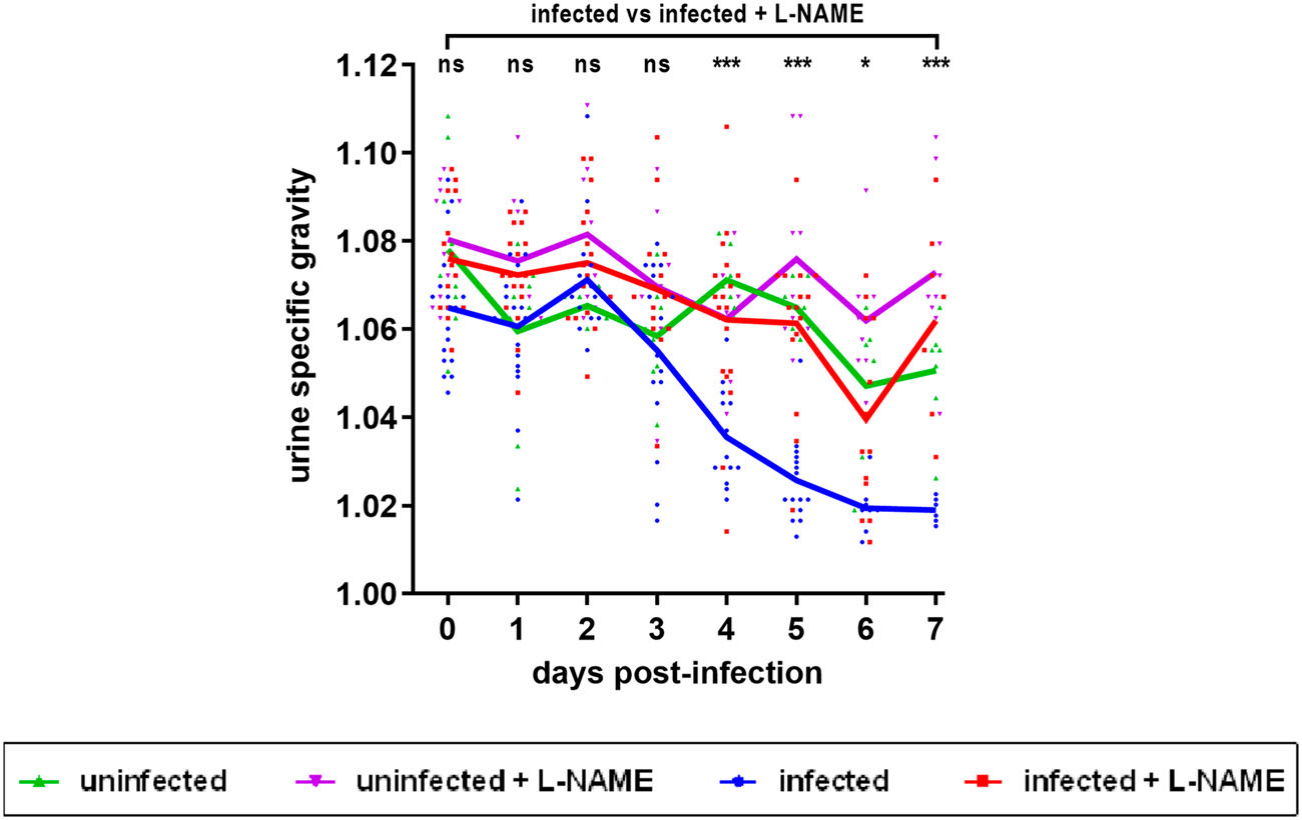
Influence of EHEC infection and L-NAME treatment on the urine specific gravity of mice. Mice, treated or not with L-NAME, were left uninfected or were infected with EDL933 and injections of ciprofloxacin were performed at 1, 2 and 3 DPI. At the indicated time points, urine was collected from each animal and the urine specific gravity was measured using a refractometer. Each dot represents one mouse and curves represent mean values. An ANOVA with the Holm-Sidak test was applied to compare all groups each day. ns: non-significant; *p <* *.05; ***p <* *.001.

We next investigated the role of NO in a lethal EHEC infection model. Following infection of mice treated or not with L-NAME, intraperitoneal injections of ciprofloxacin were performed in order to boost Stx production (see M&M). As expected, ciprofloxacin treatment led to a massive decrease of EDL933 in faeces (Figure 4(A)) as well as a high level of released toxin consequently to Stx prophage lytic cycle induction (compare Figures 3 and 4(B)). When compared to control mice, mice infected with EDL933 significantly lost weight, developed clinical signs of illness and eventually died (Figure 4(C,D)). As expected, the development of disease symptoms was strongly correlated with Stx activity level since mice presenting the highest Stx activity levels were the ones that were moribund and needed to be euthanized (Figure 4(B), open symbols). Addition of L-NAME to the drinking water of animals did not significantly alter the survival rate and weight loss of infected mice or the Stx activity quantified from faecal samples (Figure 4(B–D)). We also measured the urine specific gravity (USG) of each mouse daily in order to reveal potential kidney dysfunctions [28,29]. Whereas the USG was stable overtime for control mice, the USG decreased for EDL933-infected mice, starting from 3 DPI (Figure 5). Interestingly, the USG did not change for infected mice treated with L-NAME and values were significantly different compared to those from untreated infected mice between 4 and 7 DPI (Figure 5). Moreover, low USG values recorded from infected mice were correlated with low urea concentrations in urine (Supplementary Figure S3). Taken together, these data suggest an alteration of renal function in these animals.

**Figure 6.**
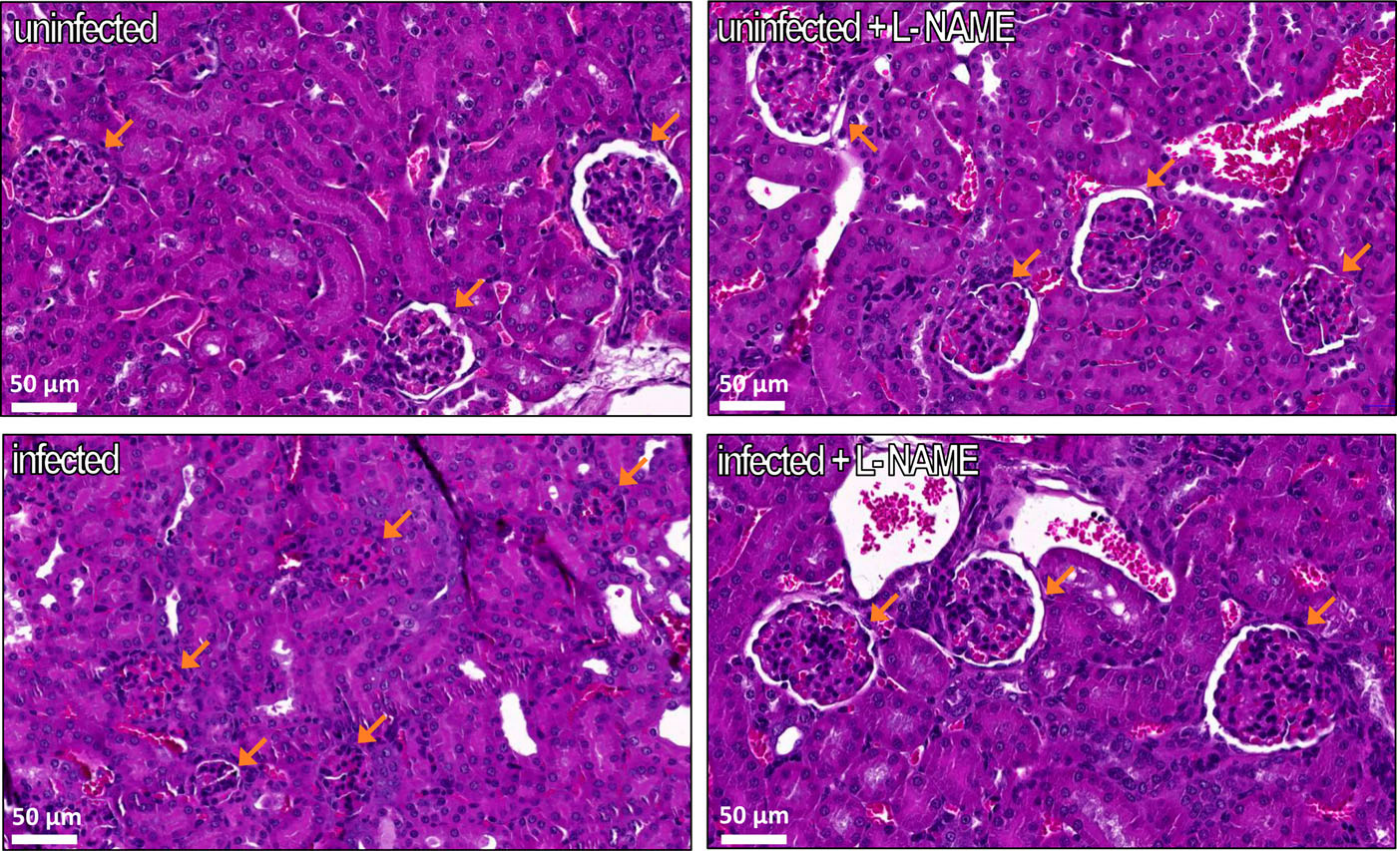
Influence of EHEC infection and L-NAME treatment on kidney structure. Mice, treated or not with L-NAME, were left uninfected or were infected with EDL933. Injections of ciprofloxacin were performed at 1, 2 and 3 DPI and mice were euthanized at 7 DPI. Kidneys were immediately harvested and organ sections were stained with hematoxylin and eosin. Representative images of the kidney of one mouse from each group are shown. Orange arrows show renal corpuscles, which are constituted of a glomerulus surrounded by the Bowman’s capsule.

**Figure 7.**
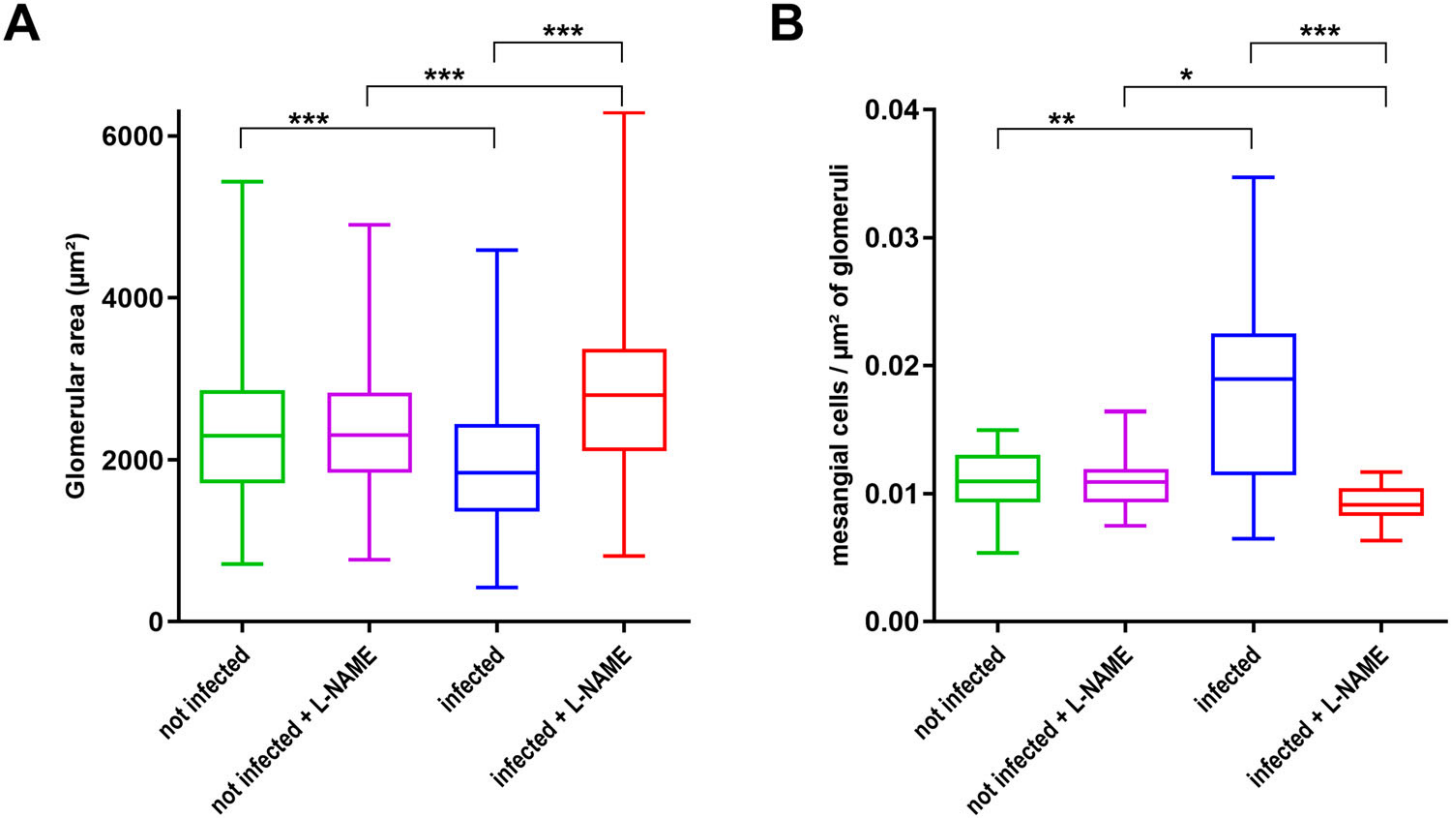
Influence of EHEC infection and L-NAME treatment on the size of glomeruli and concentration of mesangial cells within glomeruli. Mice, treated or not with L-NAME, were left uninfected or were infected with EDL933. Injections of ciprofloxacin were performed at 1, 2 and 3 DPI and mice were euthanized at 7 DPI. Kidneys were immediately sampled and organ sections were stained with hematoxylin and eosin. (A) The surface of each glomerulus observed in a kidney section was quantified from 2 to 4 mice of indicated groups (number of analysed glomeruli > 370 for each group). (B) The concentration of mesangial cells within glomeruli was calculated from 2 to 4 mice of indicated groups. Thirty glomeruli were arbitrarily selected from kidney sections and the number of mesangial cells was divided by surface of the glomerulus. Data are presented as a box and whisker plot. A Kruskal-Wallis test was applied to compare all groups. *p <* *.05; **p <* *.01; ***p <* *.001.

We next selected 2–4 mice in each group to perform histological analyses of kidneys. These mice were representative of their group with respect to most parameters mentioned above (Supplementary Figure S4). Significant structural changes, particularly in the renal corpuscles, which are units of blood filtration in kidneys, were observed between mice groups. First, most Bowman’s capsules appeared of reduced size, and sometimes were even invisible in infected mice (Figure 6). In addition, the glomeruli appeared smaller in this group of mice when compared to uninfected mice and this was confirmed by a quantitative analysis (Figure 7(A)). We also analysed the structure of kidneys in mice treated with L-NAME. Treatment of uninfected mice with L-NAME did not alter the overall structure of kidneys. However, the surface of glomeruli was increased in infected mice treated with L-NAME versus infected mice (Figures 6 and 7(A)). We also noticed a significant change in the number of mesangial cells, which affect glomerular filtration rate through their contractile activity, within the glomeruli. Indeed, we observed significantly more mesangial cells per µm^2^ of glomerulus in infected mice compared to the other groups, including infected mice treated with L-NAME (Figure 7(B)). The structural alterations of renal corpuscles in infected mice combined with the decrease of USG and urine urea concentration demonstrate that EDL933-infected mice developed a renal failure. Moreover, we observed that inhibition of NOS activity by L-NAME protects infected mice from renal damages.

## Discussion

The aim of this study was to evaluate the potential role of NO in the control of an EHEC infection. NO is an important player of the immune system, notably via its contribution to host defense against infectious agents [30]. Indeed, the antimicrobial action of NO has been well described for phagocytic cells such as macrophage in the context of the phagolysosomal pathway [31]. Once produced by intestinal cells, NO can also diffuse to the gut lumen, sometimes at high concentration like during infectious gastroenteritis [32–34]. This is in accordance with our data demonstrating that EHEC detect NO in the mouse gut as early as the first day of infection. To our knowledge, this is the first time that detection of NO by an intestinal extracellular pathogen has been demonstrated. Taken together with previous works demonstrating that several NO-regulated genes are induced during infection [23,35], these data argue that EHEC sense NO in the gut and adapt its gene expression consequently. Nevertheless, the antimicrobial impact of extracellular NO on gut bacteria and particularly on pathogens is poorly known and no data exists in the case of EHEC. For the highly related bacterium *Citrobacter rodentium*, a natural mouse pathogen eliciting attaching and effacing lesions similarly to EHEC and enteropathogenic *E. coli* (EPEC), intestinal epithelial cells produce high amount of NO in response to infection. If this immune response limits the burden of *C. rodentium* in the gut and helps the host to clear the pathogen [36,37], the contribution of extracellular NO has not been directly determined. Another study has also suggested a potential antimicrobial activity of NO within the gut by showing that NOS and NADPH oxidase are required to maintain homeostasis of gut microbes in the ileum of mice through production of RNS and ROS [38]. In addition to its antimicrobial activity, NO also alters the synthesis level of virulence factors in some pathogens through inactivation of virulence-associated transcription factors, especially iron-containing proteins [39]. In EHEC, we have demonstrated *in vitro* that NO inhibits the synthesis of two critical virulence factors, Stx2 and T3SS via the NO-sensing regulator NsrR [19,20]. Expression of others virulence determinants is probably also affected by NO since they are controlled by regulatory circuits that can be disturbed following NO exposure [40–42].

In this study, we evaluated the physiological consequences of NOS inhibition in EHEC-infected mice at different levels. While no difference was observed in bacterial shedding, the proportion of mucosa-adherent EHEC was significantly increased in the colon of mice treated with L-NAME at 7 DPI. This anti-adhesion property of NO is in accordance with our previous study which determined that chemical or cellular sources of NO inhibits EHEC adhesion to intestinal epithelial cells grown *in vitro* [20]. In addition, NO seems to have general anti-bacterial adhesion properties as determined by its efficiency to limit adhesion of several Gram-positive and Gram-negative bacteria to abiotic surfaces [43]. We also quantified the impact of NOS inhibition on Stx activity in the gut of infected mice. Until day 5, mouse treatment with L-NAME led to a significant decrease of Stx cytotoxic activity recorded from faecal samples. These results were unexpected since our team demonstrated that NO inhibits Stx2 synthesis during standard *in vitro* growth conditions [19]. This discrepancy can arise from the highly variable environmental conditions between experiments. Notably, a recent study demonstrated that NO enhances the production of Stx1 and Stx2 in EHEC grown under *in vitro* anaerobic conditions in a RecA-and Fur-dependent way [44]. Because the gastrointestinal tract is an anaerobic milieu, it may thus imply that NO would increase the synthesis of Stx1 and Stx2 in the gut of infected mice, and explain why we observed an inhibition of Stx toxicity following L-NAME treatment.

To define the potential role of NO in EHEC-associated symptoms, we also used a lethal model of infection consisting of ciprofloxacin injections to EHEC-infected mice in order to boost Stx phage induction and toxin release. In this model, the renal function was seriously affected in EHEC-infected mice, as determined by USG values and histological damages observed in kidney. Interestingly, L-NAME treatment prevented renal failure in infected mice, suggesting that NO production in response to infection can be detrimental to the host. As observed in the non-lethal mouse model of infection, NO might have increased Stx synthesis in the gut to levels sufficient to provoke a renal failure. However, we were not able to reproduce this statement at a significant level in infected-mice treated with ciprofloxacin, probably as a result of saturated induction of Stx phage lytic cycle under our experimental conditions. Another possible explanation could be linked to a better translocation of Stx across the gut mucosa in the presence of NO. Indeed, it has been reported that NO contributes to an intestinal barrier dysfunction in rodents via an increase of intestinal permeability [45,46]. We also cannot exclude the hypothesis that NO worsens the alteration of renal function in the presence of Stx since it is a well-known player acting on blood pressure as well as on renal excretory functions [47,48]. NO is produced from L-arginine by three NO synthases (NOS1, NOS2 and NOS3) and NOS2 is responsible for high level of NO production under pathophysiological conditions such as an infection [49]. As observed for other gut pathogens such as *C. rodentium* or *Salmonella enterica* serovar Typhymurium [37,50], the expression of NOS2 is induced early in EHEC-infected mice (supplementary Figure S5) and this probably contributes to NO release within the gut as detected by our NO sensing strain. We also evaluated the expression of each NOS gene in the gut of mice presenting symptoms but no significant difference were observed between groups of mice in our lethal model of infection (supplementary Figure S6). NOS2 was not up-regulated in this case probably because EHEC were cleared from the gut early in the infectious process by ciprofloxacin treatment.

Altogether, our results demonstrate that the production of NO in response to EHEC infection can be detrimental to the host, in particular to the renal function. It should be noted, however, that our mouse models of infection include treatment of animals with streptomycin and/or ciprofloxacin. Because both antibiotics affect the gut microbiota composition, NO balance in the gut has been probably also altered by these treatments. An interesting alternative to study EHEC pathogenesis without the need to treat animals with antibiotics is to infect mouse with the highly related pathogen *C. rodentium*. Despite the availability of a Stx-producing *C. rodentium* strain [51], we did not consider this model in our study because NO-dependent regulation of virulence genes has been shown to be differential between EHEC O157:H7 and *C. rodentium* [20]. Keeping in mind the limitations of mouse models to study EHEC infection, we should remain cautious about the role of NO in the outcome of an EHEC infection in human. Nevertheless, it is tempting to imagine that variations in the level of NO produced by individuals in response to infection (as a consequence of NOS polymorphism for example [52]), may explain at least partially why some but not all infected patients develop life-threatening diseases such as HUS. The role of NO against infectious agents has been investigated in other studies. Whereas NO has usually antimicrobial properties *in vitro*, the effect of NO in controlling pathogens *in vivo* seems far more complex, as exemplified by our study. While a NOS2-dependent production of NO has been shown to protect mice from several pathogens such as *Listeria monocytogenes* or *Leishmania major* [53,54], NOS2 expression has no discernible protective effect and can even worsen the disease symptoms for other infectious agents. In *Salmonella* infections, the NOS2 gene expression is highly induced in tissue of infected mice [50] but the NO subsequently produced appears to have contradictory effects. Whereas NO limits the colonization of Peyer’s patches by the pathogen as well as its replication in the spleen and liver [55,56], NO and the ensuing proinflammatory cascade also promote the fitness of *Salmonella* and allow the pathogen to outcompete the intestinal microbiota [57,58]. In neonatal meningitis caused by *E. coli*, NOS2-dependent NO has serious detrimental impact on the outcome of infection, since it promotes development of bacteremia as well as disruption of the blood–brain barrier [59,60]. Because NO is a highly reactive molecule and has pleiotropic effects on both the host and the incoming pathogen, the contribution of NO to infectious disease progression appears to be very complex and dependent of the context. In the case of EHEC, our study showed that production of NO by infected mice exacerbates the severity of symptoms and leads to kidney failure. This work pushes forward an essential role of NO-EHEC interplay in the outcome of an infection and may assist future works to evaluate the efficiency of novel therapeutic strategies based on the modulation of NO concentration by either synthesis, delivery or scavenging.

## Supplementary Material

Supplemental Material
