# Supplementary material for: Interplay between enterohaemorrhagic Escherichia coli and nitric oxide during the infectious process
Source: Emerg Microbes Infect. 2020 May 27;9(1):1065–76. doi: 10.1080/22221751.2020.1768804 (PMC7336997; doi:10.1080/22221751.2020.1768804)
Supplement: Supplemental Material [file TEMI_A_1768804_SM3415.docx]

**SUPPLEMENTARY MATERIALS**

**
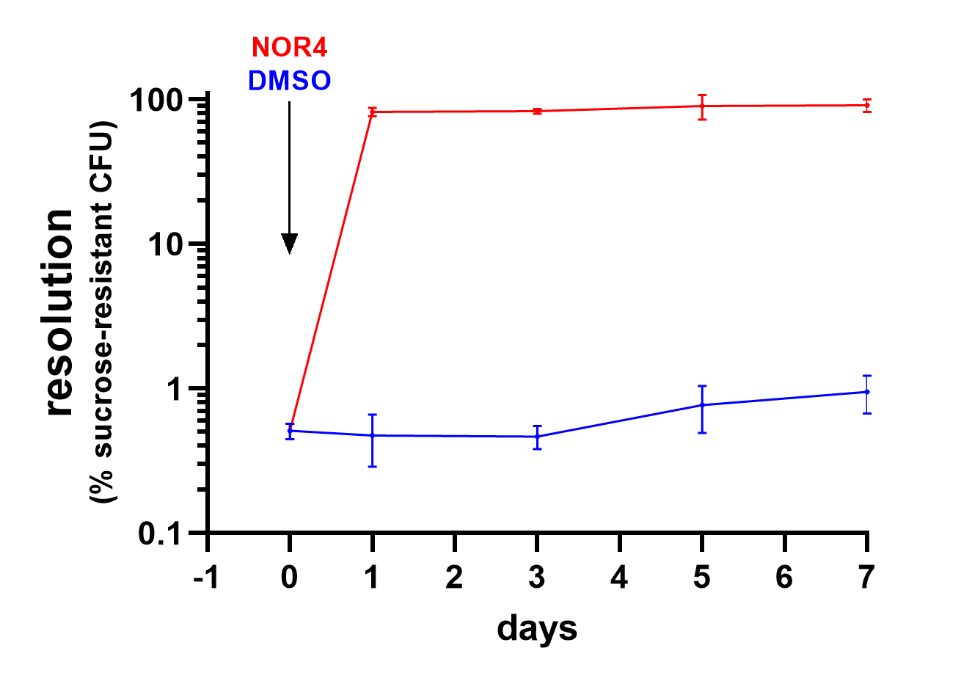
**

**Supplementary Figure S1:** The NO reporter strain was grown for 7 days in LB through successive 24 h cultures each diluted to 1/1000. The first culture (day 0) was exposed to 500 µM of NOR4 or to the equivalent volume of DMSO as a negative control. At indicated time points, resolutions (percentage of bacteria that have lost the RES marker cassette) were calculated following bacterial numeration on plates with or without sucrose. Values represent the mean +/- standard deviation.


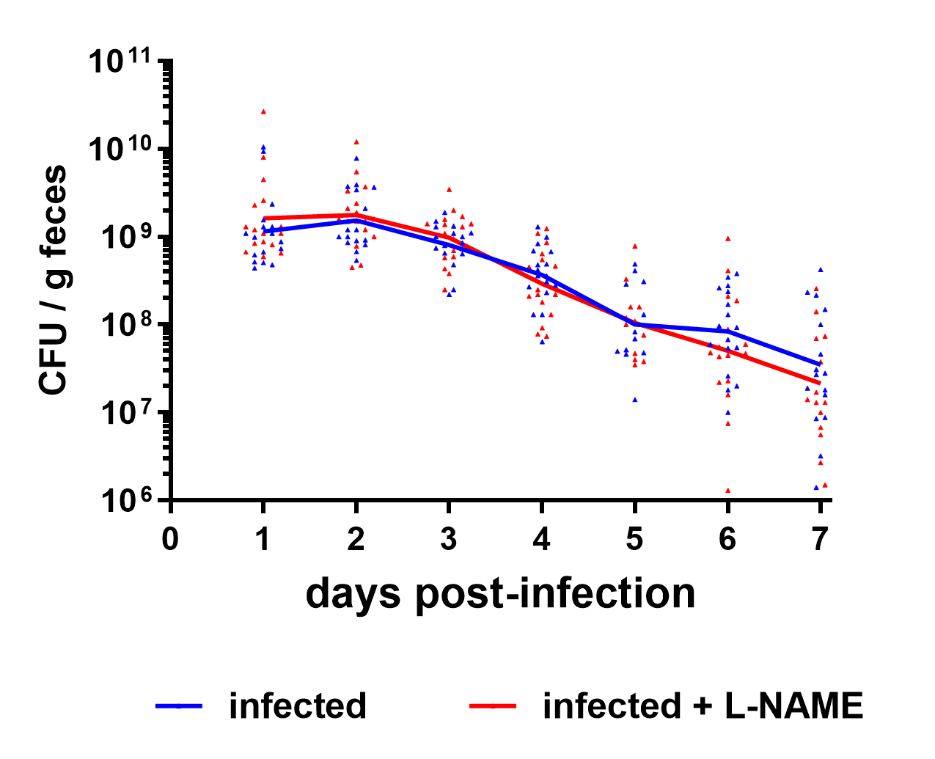


**Supplementary Figure S2:** Mice, treated or not with the NOS inhibitor L-NAME, were infected with the EHEC NO reporter strain. At the indicated time points, EHEC shedding was quantified by plating fecal samples on LB plates with Sm. Each dot represents one mouse and curves represent mean values.


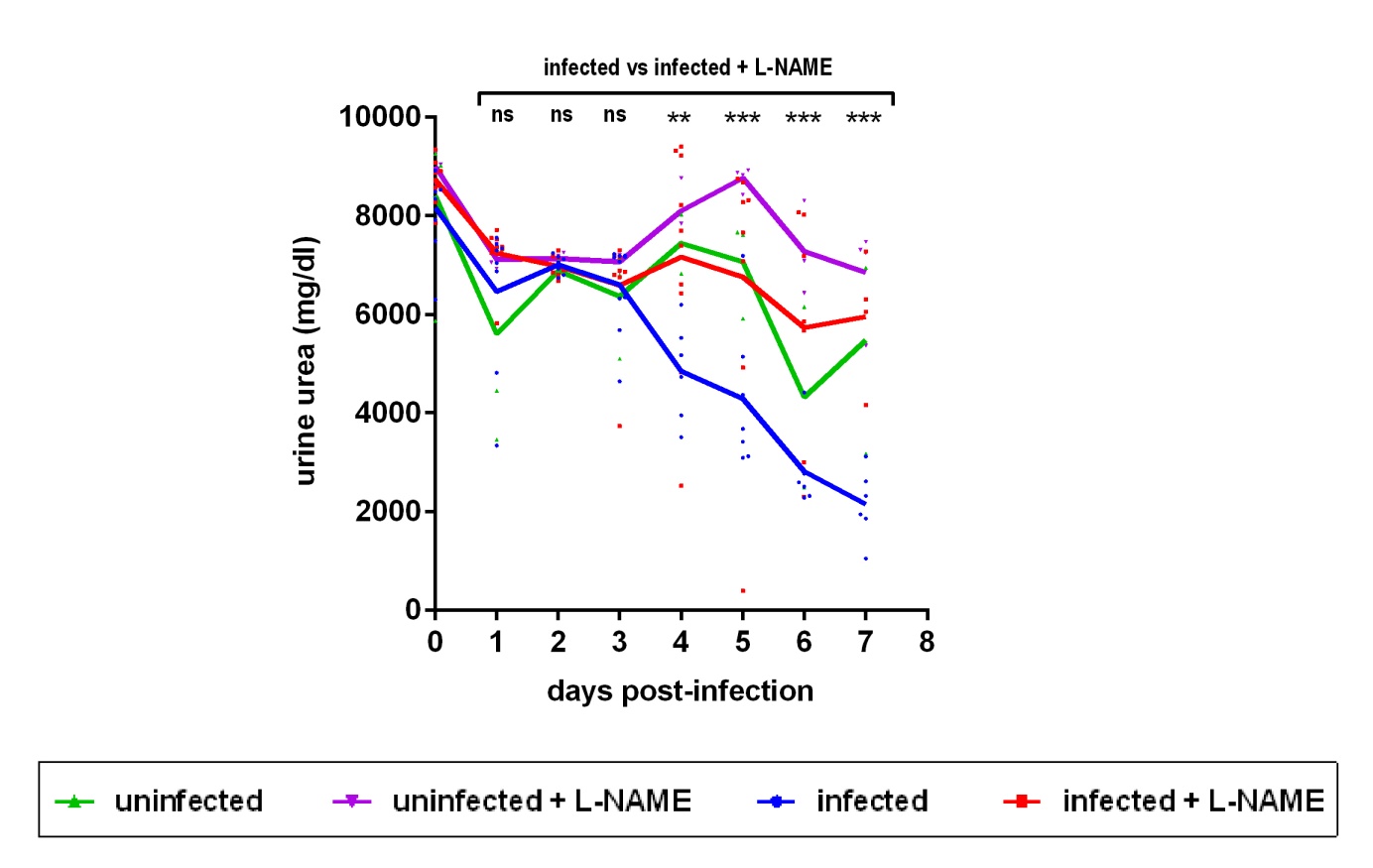


**Supplementary Figure S3:** Mice, treated or not with L-NAME, were left uninfected or were infected with EDL933. At the indicated time points, urine was collected from each animal and urine urea was quantified. Each dot represents one mouse and curves represent mean values. An ANOVA with the Holm-Sidak test was applied to compare all groups each day. ns: non-significant; ** P<0.01; *** P<0.001.


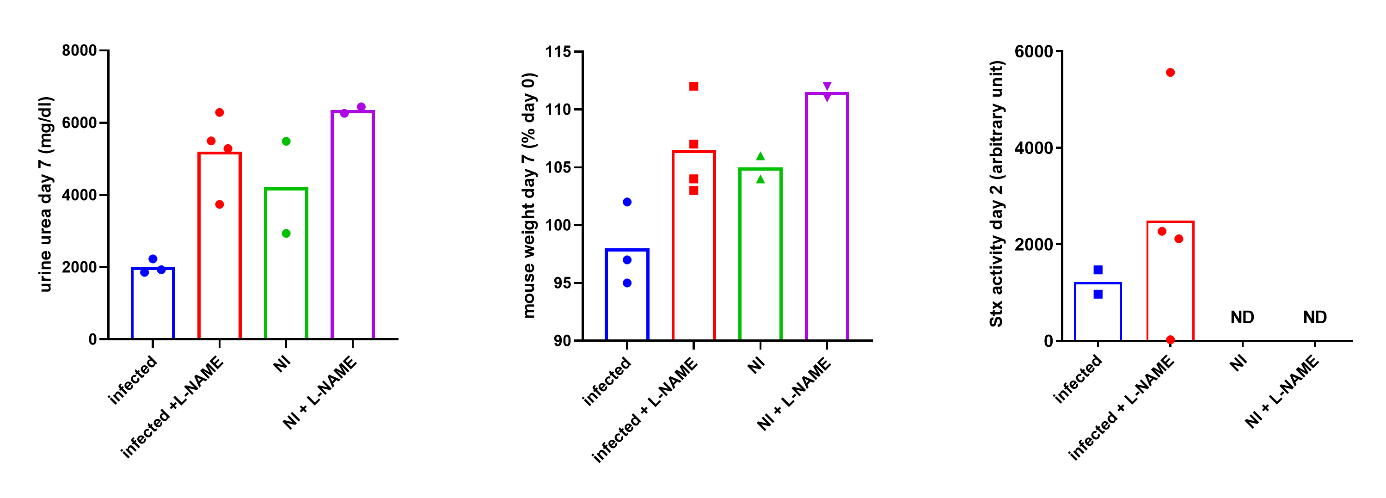


**Supplementary Figure S4:** Urine urea concentration, weight loss and fecal Stx activity recorded from mice selected for histological analyses are shown. Each dot represents one mouse and histogram represent mean values. ND: not determined


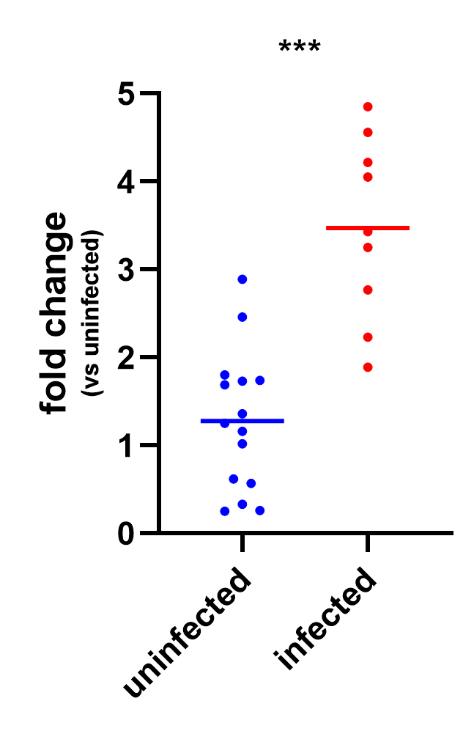


**Supplementary Figure S5:** Mice were left uninfected or were infected with EHEC EDL933. At 2 DPI, mice were euthanized and a fragment of colon was collected for RNA extraction. Expression level of NOS2 gene was quantified by qRT-PCR and β-actin was used for normalization. Results are expressed as relative mRNA expression compared with the uninfected condition. Each dot represents one mouse and means are indicated as a line. A two-tailed unpaired t-test was applied to compare both groups. *** P<0.001.


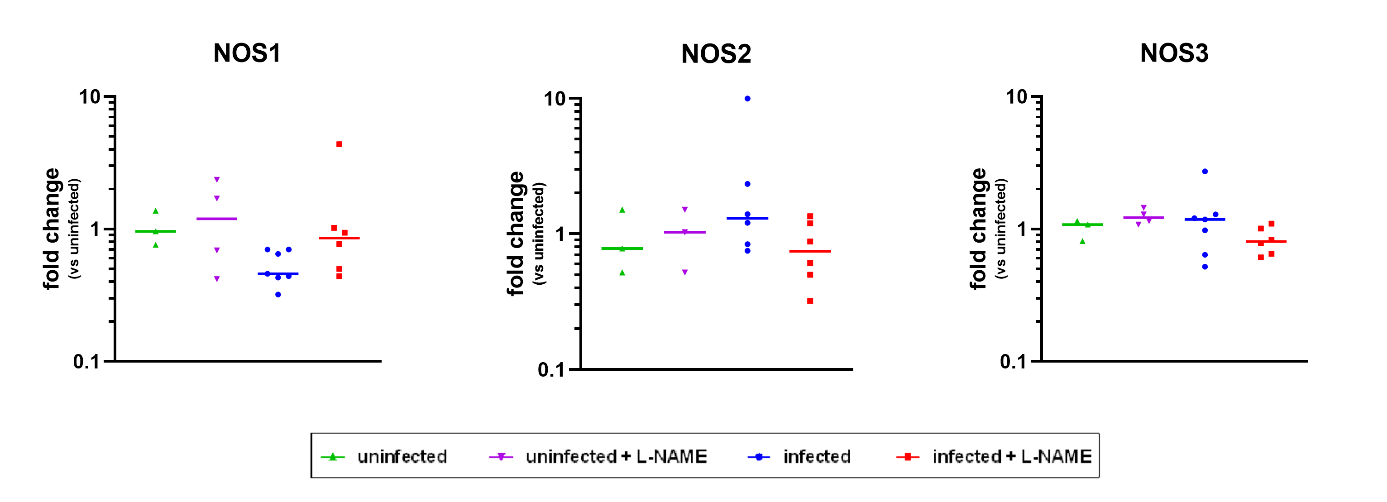


**Supplementary Figure S6:** Mice, treated or not with L-NAME, were left uninfected or were infected with EDL933. Each mouse was injected 40 µg of ciprofloxacin intraperitoneally at 1, 2 and 3 DPI to induce Stx production and release. At euthanasia (7 DPI), a fragment of colon was collected for RNA extraction. Expression level of NOS1, NOS2 and NOS3 genes was quantified by qRT-PCR and β-actin was used for normalization. Results are expressed as relative mRNA expression compared with the uninfected condition. Each dot represents one mouse and means are indicated as a line. An ANOVA with the Holm-Sidak test was applied to compare all groups. No significant difference was observed.

**Supplementary Table S1:** Bacterial strains and plasmids used in this study.

| Strain or plasmid | Description | Source or reference |
| --- | --- | --- |
| **Strains** |  |  |
| EDL933 Sm^R^ | Streptomycin-resistant derivative of EDL933 | [[1](#_ENREF_1)] |
| EDL-RES | EDL933-Sm^R^ containing *res-kan-sacB-res* cassette, Sm^R^, Kan^R^, Suc^S^ | [[1](#_ENREF_1)] |
| EDL-RES P*_ytfE_*-*tnpR* | EDL-RES carrying a P*_ytfE_*-*tnpR* fusion; Sm^R^ Kan^R^ Gm^R^ Amp^R^ | this study |
|  |  |  |
| **Plasmids** |  |  |
| pSTNSK-Cm | *oriSC101*(Ts) *tnsABCD*; Km^R^, Cm^R^ | [[2](#_ENREF_2)] |
| pGP-Tn7-Gm | *oriR6K mobRP4 Tn7-Gm; Ap^R^ Gm^R^* | [[2](#_ENREF_2)] |
| pGOA1193 | *oriR6K mobRP4 lacZ tnpR*; Ap^R^ | [[3](#_ENREF_3)] |
| p1193-*ytfE* | pGOA1193 P*_ytfE_*-*tnpR*; Ap^R^ | this study |

1. Gardette M, Le Hello S, Mariani-Kurkdjian P, et al. Identification and prevalence of *in vivo*-induced genes in enterohaemorrhagic *Escherichia coli*. Virulence. 2019 Dec;10(1):180-193.

2. Crepin S, Harel J, Dozois CM. Chromosomal complementation using Tn7 transposon vectors in *Enterobacteriaceae*. Appl Environ Microbiol. 2012 Sep;78(17):6001-8.

3. Osorio CG, Crawford JA, Michalski J, et al. Second-generation recombination-based *in vivo* expression technology for large-scale screening for *Vibrio cholerae* genes induced during infection of the mouse small intestine. Infect Immun. 2005 Feb;73(2):972-80.
